# The erythrocyte sedimentation rate in male adolescents and subsequent risk of Parkinson’s disease: an observational study

**DOI:** 10.1007/s00415-020-10324-5

**Published:** 2020-12-04

**Authors:** Camilla Fardell, Linus Schiöler, Hans Nissbrandt, Kjell Torén, Maria Åberg

**Affiliations:** 1 Department of Pharmacology, Institute of Neuroscience and Physiology, The Sahlgrenska Academy, University of Gothenburg, Gothenburg, Sweden; 2 Occupational and Environmental Medicine, School of Public Health and Community Medicine, Institute of Medicine, The Sahlgrenska Academy, University of Gothenburg, Gothenburg, Sweden; 3 Department of Occupational and Environmental Medicine, Sahlgrenska University Hospital, Gothenburg, Sweden; 4 School of Public Health and Community Medicine/Primary Health Care, Institute of Medicine, The Sahlgrenska Academy, University of Gothenburg, Box 454, 405 30 Gothenburg, Sweden; 5 Region Västra Götaland, Regionhälsan, Gothenburg, Sweden

**Keywords:** Parkinson’s disease, Inflammation, Erythrocyte sedimentation rate, Adolescence, Population

## Abstract

Systemic inflammation may be implicated in the pathophysiology of Parkinson’s disease (PD). Since PD occurs usually in later life, most studies of causal factors are conducted in older populations, so potentially important influences from early life cannot be adequately captured. We investigated whether the erythrocyte sedimentation rate (ESR) in early adulthood is associated with the subsequent development of PD in men. As part of Swedish national conscription testing conducted from 1968 through 1983 (*N* = 716,550), the erythrocyte sedimentation rate, as a measure of inflammation, was measured in 659,278 young men. The cohort was observed for subsequent PD events (*N* = 1513) through December 2016. Cox proportional hazards models were used to estimate the hazard ratios (HR) with 95% CI with adjustment for potential confounders. Individuals with higher ESRs were significantly less likely to be diagnosed with PD, as ESR was linearly and inversely associated with PD risk. The magnitude of the association between ESR and PD risk was similar for increases up to 15 mm/h, leveled off thereafter, and was non-significant for ESR values > 20 mm/h. The HR for PD with basic adjustments (age at conscription, year of conscription, test center and erythrocyte volume fraction) was 0.94 (95% CI 0.89–0.99, *P* = 0.02) per log_2_ increase in ESR, corresponding to a two-fold increase in ESR. Further adjustments for potential confounders (parental education, systolic and diastolic blood pressures, and IQ) scarcely altered the HR. The results suggest a prospective association between high ESR and reduced risk for PD.

## Introduction

Parkinson´s disease (PD) is a progressive neurodegenerative disease that is characterized by bradykinesia, rigidity, and tremors, mainly due to the death of dopaminergic neurons in the substantia nigra [1]. Although environmental and genetic risk factors have been implicated in PD, the etiology is not fully understood.

Neuroinflammation is a feature of PD pathology. Autoreactive T lymphocytes, autoantigen presentation, increased levels of inflammatory cytokines in the brain and cerebrospinal fluid, and microglial activation have been observed in patients with PD and in animal models of the disease [2–7]. Recent observations suggest a role for gastrointestinal inflammation in the initiation and progression of PD [8]. Moreover, several studies have shown a significant association between the levels of pro-inflammatory markers and PD severity [9–12].

In contrast, conditions associated with low-grade systemic inflammation, such as smoking and hyperuricemia, have been shown to be protective against PD [13–16]. In a recent cohort study, increasing concentrations of systemic inflammation markers during a 20-year period prior to diagnosis were associated with a lower risk of PD [17]. Thus, it remains to be determined whether neuroinflammation promotes or protects against neurodegeneration. Inflammatory processes seem be involved in the pathophysiology of PD, although whether these are causal or a consequence of the progression of PD is unclear.

Prospective longitudinal studies are needed to uncover whether changes in inflammatory markers precede an increased or a decreased risk of developing PD. The erythrocyte sedimentation rate (ESR) is a non-specific marker of inflammation [18, 19]. It reflects a systemic response to inflammation, and is related primarily to the blood concentrations of fibrinogen and immunoglobulin [20]. When inflammation is present, fibrinogen causes the erythrocytes to clot in rolls, which means that they sink faster in a test tube, resulting in an increased ESR. ESR is a simple and cost-efficient way of establishing indirectly the activities of pro-inflammatory cytokines [21], making it a suitable marker of inflammation when examining large cohorts.

The objective of this study was to examine whether the ESR measured in young adulthood is associated with the risk of developing PD later in life. We performed an exploratory, population-based, prospective study of late-adolescent men who underwent medical examinations upon compulsory military conscription.

## Subjects and methods

### Study population

The present study is based on data retrieved from the Swedish Military Service Conscription Registry. The cohort considered for inclusion in this study comprised all Swedish men (born between 1951 and 1965) who were conscripted for compulsory military service between 1969 and 1983 (*N* = 716,550). During this period, exemptions from military conscription for men in Sweden were given only to those who were imprisoned or suffered from a severe chronic medical condition or disability, representing 2–3% annually of Swedish men.

Since every Swedish resident is assigned a unique personal identification number, linkage of the different registers is possible. The National Cause of Death Register and the Statistics Sweden database were used to identify participants who died or emigrated during the follow-up period. Women who enlisted during this period were excluded from the study (*N* = 570). Men (*N* = 15,027) with unknown or extreme values for ESR or erythrocyte volume fraction (EVF; also known as hematocrit) (see below) at conscription were excluded, as were 26 individuals who were diagnosed with PD before conscription. Among the subjects who developed PD during the follow-up period, only those who were diagnosed at or after the age of 40 years were included in the present study. The exclusion criteria were: late allocated personal identity number (i.e., personal identity number reallocated from deceased person), > 25 years of age at conscription, missing data regarding the conscription test center, or PD diagnosis, and death or emigration before 40 years of age. In total, 659,278 conscripts were included in the present study, of which 1,513 developed PD later in life. In the period 1987–1996, the International Classification of Diseases, ninth and tenth revisions (ICD-9 and ICD-10) were in use. The cases consisted of men who had a first-time diagnosis of PD listed in the National Hospital Discharge Register or on their death certificate.

### Conscription register data

The conscripts underwent standardized physical and cognitive examinations over a 2-day period, conducted by a psychologist and a physician at one of six designated conscription centers in Sweden (Southern, Western, Eastern, Central, Northern Lower, and Northern Upper). Bodyweight was measured to the nearest kilogram and height was measured to the nearest centimeter using standardized equipment. Resting systolic and diastolic blood pressures were measured.

### Blood samples

Venous samples were taken at conscription for analysis of the ESR and EVF. The ESR is defined as the distance that a column of anticoagulated blood falls in 1 h. The EVF is defined as the ratio of the volume occupied by red blood cells relative to the volume of whole blood. The ESR was measured according to standard laboratory procedures using the Westergren method [18]. The EVF analyses were performed by the microhematocrit method and were consistent with the National Committee for Clinical Laboratory Standards. As described in the “Statistical analysis” section, analyses involving ESR were adjusted for EVF.

### Parental education level

Information on the level of education of the conscripts´ parents were obtained from the longitudinal integration database for Health Insurance and Labour Market Studies (LISA, 80% coverage). This database, which was started in 1990 and includes all registered residents aged 16 years and older, integrates data from the labor market and educational and social sectors. In the analysis, parental education level was assigned to one of three categories: pre-high school education for up to 9 years (low); high school education for up to 12 years (medium); and university education for ≥ 2 years and postgraduate training (high). The highest level of education attained by either parent was used in the analyses.

### IQ measurements

The cognitive abilities were assessed at conscription using the Swedish Enlistment Battery (SEB). Two different versions of the SEB, SEB67 and SEB80, were used during the study period. These consisted of four sub-tests that measured verbal, logical, visuospatial, and technical capabilities, the scores for which were then summed to generate the Global IQ score. The outcome of each sub-test and the Global IQ score were ranked on a scale of 1–9, with 1 being the lowest scoring group and 9 the highest scoring group. The nine points on the scale correspond approximately to IQ bands < 74, 74–81, 82–89, 90–95, 96–104, 105–110, 111–118, 119–126, and > 126, respectively [22].

SEB67 was used during the period 1968–1979. “Instructions” was a logical test comprising 40 items that measured the ability to understand instructions and apply them to solve a problem. “Concept Discrimination” consisted of 40 items and examined verbal ability by having the conscript choose which word out of five alternatives did not agree with the others conceptually. “Paper Form Board” was a visuospatial test with 25 items, containing questions about two-dimensional puzzles. “Technical Comprehension” was a 52-item test comprising illustrated technical and physical problems. SEB80 was used during the period 1980–1993. “Instructions” was retained with some improvements. “Concept Discrimination” was replaced by “Synonyms”, which involved 40 items to assess vocabulary by letting the conscript identify which of four alternatives was the synonym of a given word. “Paper Form Board” was replaced by another visuospatial test, “Metal Folding”. This 40-item test measured geometrical perception by having the conscript identify the correct three-dimensional object from a series of two-dimensional drawings. “Technical Comprehension” was modified to measure knowledge of mathematics, physics and chemistry, consisting of 40 items.

### Smoking

Data on smoking habits at baseline were available for a sub-cohort of 21,846 participants conscripted in the period 1969–1970. Apart from the standard physical and cognitive examinations, the 1969–1970 conscripts participated in a survey that was designed to collect information on social background, behavior and social adjustment, and substance use, e.g., alcohol consumption and tobacco smoking. The conscripts were asked to report their smoking habits according to one of the following five levels: non-smoker, 1–5 cigarettes per day, 6–10 cigarettes per day, 11–20 cigarettes per day, and > 20 cigarettes per day.

### Outcomes

#### Data sources

Data from the Swedish Military Service Conscription Register were linked to the Swedish Hospital Discharge Register and the Swedish Cause of Death Register. The Swedish Hospital Discharge Register was initiated in 1968 and the coverage was gradually increased until 1986; it is complete since 1987. Diagnoses made in hospital outpatient settings have been recorded since 2001. Deaths were identified by linkage with the Swedish Cause of Death Register, which is updated annually based on the issuance of death certificates and covers virtually all deaths since 1961.

#### PD diagnoses

PD diagnoses in the study cohort through December 31, 2016 were acquired using the conscripts´ personal identification numbers from the Swedish Hospital Discharge Register or the Swedish Cause of Death Register. Between 1987 and 2016, the International Classification of Diseases, ninth and tenth revisions (ICD-9 and ICD-10) were in use. Diagnoses of PD were coded as 332A in records using the Swedish version of the ICD-9 (1987–1997) and as G20 in records using the ICD-10 (1998–2016). The Swedish Hospital Discharge Register is administered by the Center for Epidemiology of Sweden’s National Board of Health and Welfare. Cases consisted of men who had a first-time diagnosis of PD in the National Hospital Discharge Register or on their death certificate.

### Statistical analysis

Cox proportional hazards models were used to estimate hazard ratios (HRs) with 95% CI, to assess the influences of presumptive predictors on the diagnosis of PD. Tests based on scaled Schoenfeld residuals showed no significant violations of the proportional hazards assumption. The follow-up period started at the date of conscription (baseline). Subjects were followed until the time of: (1) first hospitalization for PD or hospital-based outpatient clinic contact for PD; (2) death; (3) emigration; or (4) the end of follow-up, on December 31, 2016 (minimum of 15 years and maximum of 48 years of follow-up).

The distribution of the ESRs was skewed to the right, so a logarithmic transformation was performed. In additional models, ESR was modeled as a restricted cubic spline. Due to skewness, the knots were placed at 4, 9 and 14, corresponding to the 75th, 95th and 98th percentiles among the cases of PD. As the number of red blood cells influences the ESR, the EVF was used as a covariate in the Cox regression model involving ESR. EVF and systolic and diastolic blood pressures were adjusted for using cubic restricted splines with knots placed at the 10th, 50th and 90th percentiles. Global IQ scores were categorized as: low (stanine score 1–3); medium (4–6); and high (7–9), with the latter used as the reference. Parental education level was trichotomized (low, medium, and high), as described earlier. Conscription year and test center were adjusted for in all the regression models, to account for temporal differences and procedural differences across the participating sites. A cubic restricted spline with knots at the 5th, 35th, 65th and 95th percentiles was used for conscription year. Furthermore, a second-degree polynomial was used to model age at conscription, due to the limited age range.

In Model 1, the association of ESR with risk of developing PD was assessed with adjustments for: age at conscription, year of conscription, test center, and EVF. Model 2 had additional adjustments for parental education level and systolic and diastolic blood pressures. Model 3 was additionally adjusted for Global IQ score.

Sensitivity analyses regarding incident inflammatory diseases were performed by excluding men who were diagnosed with inflammatory bowel disease (diagnostic codes 563 and 555–556 in ICD-8; ICD-9) or rheumatoid arthritis (712,00–712,39; 714 in ICD-8; ICD-9) on any occasion before conscription and up to 5 years after conscriptions.

The statistical calculations were performed with the SAS ver. 9.4 software (SAS Institute, Cary, NC, USA).

The Ethics Committee of the University of Gothenburg and Confidentiality Clearance at Statistics Sweden approved the study. The requirement for informed consent was waived for the current study as it was a secondary analysis of existing data. The investigation conforms to the principles outlined in the Declaration of Helsinki in relation to ethical principles for medical research involving human subjects.

## Results

**Fig. 1:**
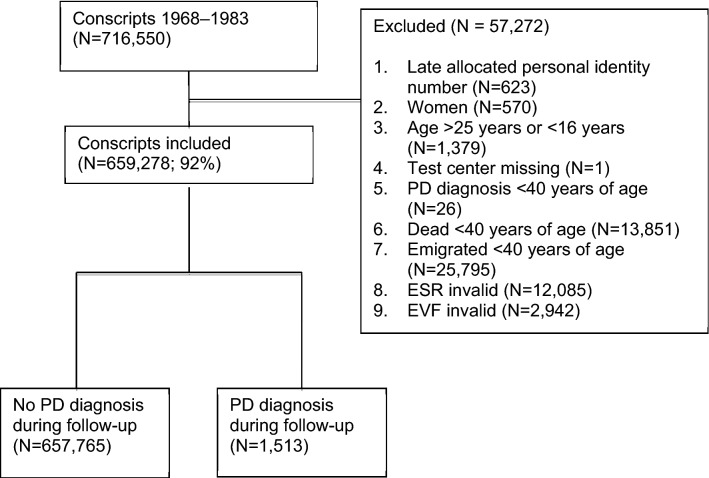
Flow chart illustrating the exclusion criteria, number of diagnoses of Parkinson´s disease (PD), and follow-up time of the Swedish male conscript study population in the period 1968–1983

The present study included 659,278 men who were followed for a total of 11,474,755 person-years from 1969 to 2016 (Fig. 1), with the analysis based on the recommendations in Strengthening the Reporting of Observational Studies in Epidemiology (STROBE) [23]. In total, 57,272 conscripts were excluded from the analyses, and 1513 were diagnosed with PD during the 48 years of follow-up. The mean (± SD) age at the end of follow-up was 57 (± 5) years. Table 1 shows the descriptive data for the conscripts according to ESR group. For the ESRs at conscription: 531,442 (80.61%) had 1–4 mm/h; 99,025 (15.02%) had 5–9 mm/h; 18,717 (2.84%) had 10–14 mm/h; and 10,094 (1.53%) had ≥ 15 mm/h. For the entire cohort, the mean age at conscription was 18.4 years and the mean ESR was 3.3 mm/h (± 3.3). The mean height of the men was 1.80 m (± 0.1) and the mean bodyweight was 68.6 kg (± 9.9), giving a mean BMI of 21.4 (± 2.7). The mean systolic blood pressure was 128.3 mmHg (± 11.1) and the mean diastolic blood pressure was 69.0 mmHg (± 9.7). Higher ESR was associated with lower IQ and a lower level of parental education, in a dose–response manner.

**Table 1:** Baseline characteristics of the study subjects according to ESR

	ESR (mm/h)			
	1–4	5–9	10–14	≥ 15
	*N* = 531,442	*N* = 99,025	*N* = 18,717	*N* = 10,094
	(80.61%)	(15.02%)	(2.84%)	(1.53%)
Age in years, mean (SD)	18.4 (0.7)	18.3 (0.7)	18.3 (0.7)	18.4 (0.7)
Height in cm, mean (SD)	1.8 (0.1)	1.8 (0.1)	1.8 (0.1)	1.8 (0.1)
Weight in kg, mean (SD)	68.7 (9.8)	68.6 (10.5)	68.7 (11.2)	67.8 (11.1)
BMI, mean (SD)	21.4 (2.7)	21.5 (2.9)	21.6 (3.1)	21.4 (3.1)
Systolic BP in mmHg, mean (SD)	128.5 (11.1)	127.8 (11.1)	127.9 (11.1)	127.5 (11.0)
Diastolic BP in mmHg, mean (SD)	69.1 (9.7)	68.6 (9.6)	68.9 (9.7)	69.4 (9.6)
Parental education, *N* (%)				
Pre-highschool (≤ 9 years)	227,816 (44.7)	44,912 (47.3)	8804 (49.3)	4918 (51.5)
Highschool (≤ 12 years)	193,045 (37.9)	34,956 (36.9)	6485 (36.3)	3419 (35.8)
University (≥ 2 years)	88,547 (17.4)	14,986 (15.8)	2559 (14.3)	1218 (12.7)
Global IQ score, ranked on a scale of 1–9, *N* (%)				
1–3	108,538 (20.4)	21,322 (21.6)	4,179 (22.4)	2420 (24.0)
4–6	286,825 (54.0)	53,627 (54.2)	10,085 (53.9)	5526 (54.9)
7–9	135,572 (25.5)	23,980 (24.2)	4431 (23.7)	2125 (21.1)
PD during follow-up, *N* (%)	1246 (0.23)	196 (0.20)	46 (0.25)	25 (0.25)
Age in years at follow-up, mean (SD)	57 (5)	58 (5)	58 (5)	58 (5)
ESR, mean (SD)	2.1 (1.0)	6.2 (1.3)	11.5 (1.4)	21.2 (8.0)
EVF, %, mean (SD)	46.7 (2.4)	45.2 (2.3)	44.9 (2.4)	44.5 (2.5)

*SD* standard deviation, *BMI* body mass index, *BP* blood pressure, *PD* Parkinson´s disease, *ESR* erythrocyte sedimentation rate, *EVF* erythrocyte volume fraction

**Fig. 2:**
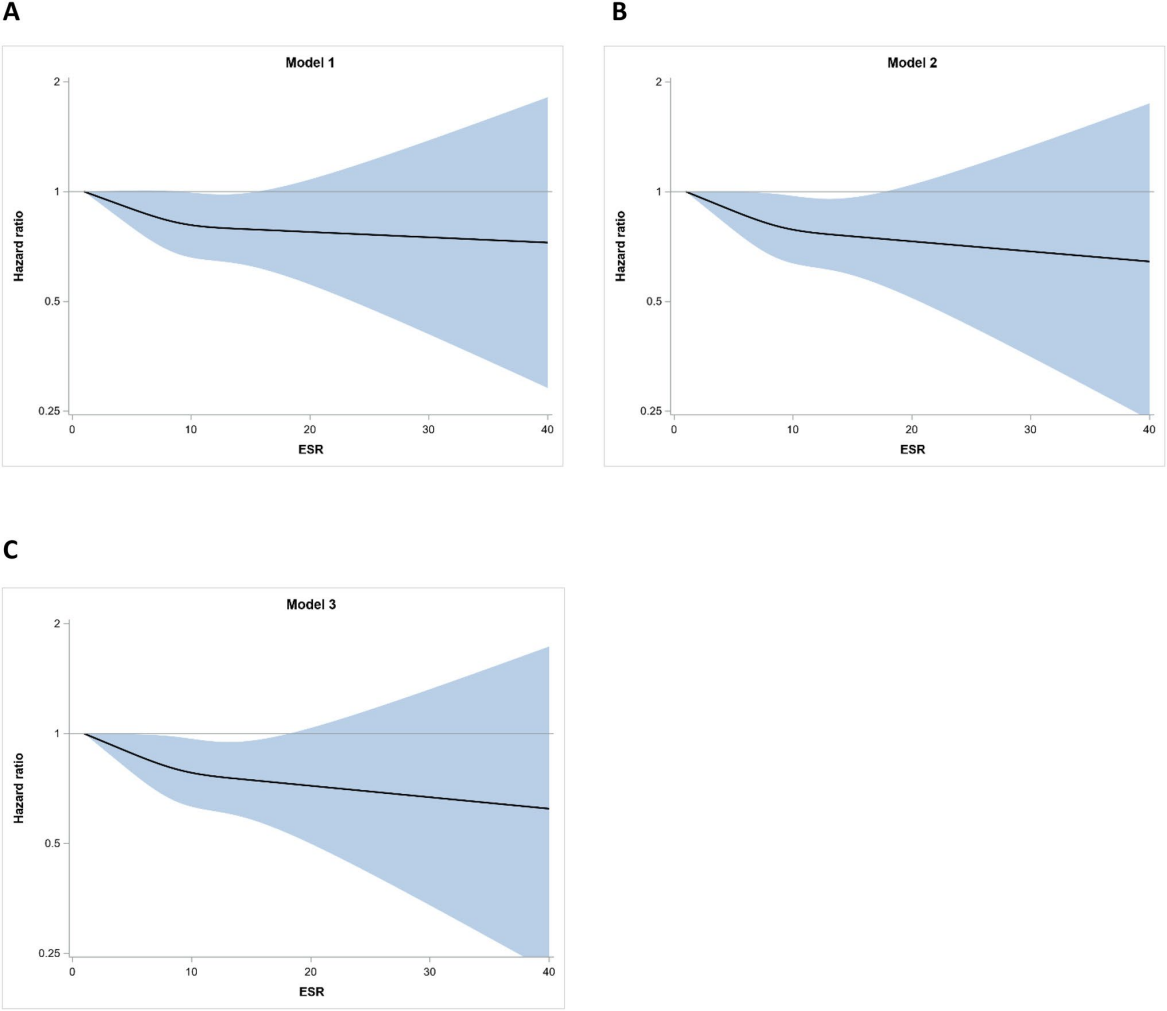
Hazard ratios (HRs) for erythrocyte sedimentation rate (ESR) at conscription and prospective risk for Parkinson´s disease (95% CI). ESR is modeled as a restricted cubic spline with knots at 4, 9 and 14. The shaded areas represent the 95% confidence intervals. **a** Basic adjustments for age at conscription, year of conscription, test center and erythrocyte volume fraction (EVF). **b** Adjustments for age at conscription, year of conscription, test center, EVF, parental education level, and systolic and diastolic blood pressures. **c** Adjustments for age at conscription, year of conscription, test center, EVF, parental education level, systolic and diastolic blood pressures, and Global IQ score

Individuals with higher ESRs were significantly less likely to be diagnosed with PD, as ESR was inversely associated with risk of PD. The hazard ratio (HR) for PD with basic adjustments (age at conscription, year of conscription, test center and EVF) was 0.94 (*P* = 0.02, 95% CI 0.89–0.99) per log_2_ increase in ESR, corresponding to a two-fold increase in ESR (Table 2). Further adjustments for potential confounders (parental education, systolic and diastolic blood pressures, and IQ) resulted in very small changes to the HR. In addition, ESR was modeled as a restricted cubic spline (Fig. 2). The association between ESR and PD risk was similar for increases in ESR up to 15 mm/h, leveled off for ESRs > 15 mm/h, and became non-significant at ESR values > 20 mm/h.

**Table 2:** Associations between ESR and the risk of PD for the study subjects, investigated using Cox proportional hazards models

	HR	95% CI	*P *value
ESR, model adjusted for:			
Age + year of conscription + test centre + EVF	0.94	0.89–0.99	0.02
+ Parental education level + systolic BP + diastolic BP	0.93	0.88–0.99	0.02
+ Global IQ score	0.93	0.88–0.99	0.01

*BP* blood pressure, *CI* confidence interval, *ESR* erythrocyte sedimentation rate, *EVF* erythrocyte volume fraction, *HR* hazards ratio, *PD* Parkinson´s disease

Sensitivity analyses in which the analyses for ESR and subsequent PD were repeated while excluding men with inflammatory bowel disease or rheumatoid arthritis (*N* = 2091) did not change the HRs.

The effect of smoking status was examined in a sub-cohort of 21,846 conscripts. The mean ESRs (± SD) for the groups of non-smokers and those who smoked 1–5, 6–10, 11–20, and > 20 cigarettes per day were 3.2 (± 3.7), 3.4 (± 4.6), 3.2 (± 3.8), 3.2 (± 4.1), and 3.1 (± 3.9), thus no significant trends.

## Discussion

The results of the present study show that individuals with higher levels of the inflammatory marker ESR are significantly less likely to be diagnosed with PD, as ESR in adolescence was found to be inversely associated with risk of developing PD in later life. The association is dose-dependent and persists after controlling for relevant confounders (parental education, systolic and diastolic blood pressures, and IQ). The splines from the present study suggest that the magnitude of the association between ESR and PD risk is similar for increases up to 15 mm/h and levels off thereafter.

### Possible mechanisms

To the best of our knowledge, this is the first study to report the association between the levels of an inflammatory marker in adolescence and the risk of PD in adulthood. Previously, we found no prospective evidence for a linkage between adolescent inflammation and the neurodegenerative disease amyotrophic lateral sclerosis (ALS) [24]. Inflammation seems to play an important role in PD progression, and there is considerable debate in the field as to whether inflammation is a primary contributor to neurodegeneration or represents a response to neuronal death. Several studies have investigated inflammatory markers in patients already diagnosed with PD [3], reporting higher peripheral inflammatory activities in the serum or plasma of patients with PD. In contrast to those studies, our results show that adolescent men with higher ESRs have a lower risk of developing PD. This in line with another recent large cohort study where PD, in contrast to ALS patients, appeared to have lower levels of circulating immune biomarkers (i.e., leukocytes and haptoglobin) 10–20 years prior to diagnosis, as compared to controls [17]. However, the statistically significant differences were only noted for parts of the respective time intervals after multivariable adjustment [17].

The ESR is a simple, non-specific screening test that indirectly measures the presence of inflammation in the body. It calculates the rate of sedimentation of red blood cells within a 200-mm vertical tube of anticoagulated blood. The process of erythrocyte sedimentation is largely influenced by the initial aggregation of red blood cells, which is facilitated by the presence of plasma proteins of high molecular weight. Thus, a high concentration of certain plasma proteins, e.g., fibrinogen and immunoglobulins, causes a high ESR [25]. The blood concentration of fibrinogen, which is an acute phase reactant, is increased in inflammatory situations [26]. It is important to note that the ESR may not correlate fully with the levels of other inflammatory biomarkers. Most studies that have addressed the association between the levels of inflammation and disease in patients with PD have defined inflammation using the plasma concentrations of C-reactive protein (CRP), interleukin-6 (IL-6) or tumor necrosis factor (TNF). Until we can identify people with PD earlier in the course of their disease, we will not be able to establish definitively whether inflammation is an etiologic factor or a resultant process in the pathophysiology of the disease.

It is possible that our results indicate that a high ESR protects against PD. Fibrinogen can activate microglia, and it has been debated whether microglial activation protects against or exacerbates neuronal loss [27]. Microglia could increase neuronal survival through the release of trophic and anti-inflammatory factors [28]. In PD, the degeneration of dopaminergic neurons is associated with microglial activation [29], which could be seen as an epiphenomenon rather than having a direct involvement in the pathogenesis of the disease. Moderately activated microglia appear to play a homeostatic role in the CNS and form the first line of defense against, for example, inflammation of the gastrointestinal tract [8], by regulating immunological and inflammatory responses [30].

An alternative explanation is that individuals who later develop PD have a weaker ability to produce a high ESR. An unpublished study from our group shows that patients with PD have weaker antibody responses to Varicella Zoster Virus, as compared to age-matched controls [31]. Additional indications of an altered immune response in PD are that patients with PD display reduced number of T-helper and B lymphocytes [32, 33]. Individuals with a reduced ability to produce antibodies should be more prone to infections. It has been shown that patients with PD have a higher infection burden [34].

Since ESR is a nonspecific inflammatory marker, one cannot exclude the possibility that other diseases might influence the ESR and confound the association with PD. In the study of Toss et al. [35], Swedish conscripts diagnosed with colitis, rheumatoid arthritis and the common cold were shown to have a significantly higher ESR, whereas conscripts diagnosed with migraine and epilepsy had a significantly lower ESR, as compared to the mean ESR for the overall cohort. Non-steroidal anti-inflammatory drugs (NSAIDs), particularly ibuprofen, may reduce the risk of developing PD [36]. Moreover, since many diseases associated with a higher ESR, such as rheumatoid arthritis, are treated with these drugs, NSAIDs may act as a mediator of the inverse association between ESR and PD. In the present cohort, we were not able to address the potentially confounding effects of anti-inflammatory treatments since the Swedish Prescribed Drug Register was initiated in 2005. We would need this information long before this time-point in order to adjust for anti-inflammatory medications during the follow-up period. A low-grade ESR increase may be a marker of early (preclinical) inflammatory disease. Previous studies have demonstrated associations between inflammatory bowel disease and increased PD risk, and between the use of immunosuppressive therapy and reduced PD risk [8, 37]. Therefore, we performed sensitivity analyses in which we repeated the analyses of the relationship between ESR level and risk of incident PD while excluding individuals with inflammatory bowel disease or rheumatoid arthritis before conscription and up to 5 years after conscription. These exclusions did not affect the associations.

It is possible that our results reflect a confounding effect of smoking [15]. Therefore, we investigated whether the ESR was associated with smoking in a sub-cohort. However, as reported previously from this cohort [35] ESR was not associated with smoking, making it unlikely that smoking is acting as a confounder in this case. However, even if the smokers on a group-level have similar ESRs as non-smokers, this does not preclude a minor or modest relationship to PD at the individual level.

In the present study, a higher ESR was associated with lower IQ and a lower level of parental education, in a dose–response manner. This may be due to socioeconomic factors, which to a large extent determine the environment of an individual, such as family size and exposure to infections. In addition, our observation of a difference in the incidence of PD in relation to high ESR/low IQ may reflect the fact that persons who fall into this category are less likely to seek early medical intervention, mainly for socioeconomic reasons. We have recently shown that high IQ appears to be a risk factor for PD [38]. However, adjustments for parental education and IQ score did not alter the association between ESR and PD.

### Strengths and limitations

The unique strength of the present study is the use of a nationwide cohort of young men. Our study sample consisted of 659,278 conscripts, encompassing the vast majority of all Swedish men born between 1951 and 1965. The large sample size facilitates a highly accurate estimation of the association between the levels of ESR and risk for PD. Having a study sample that represents such a large percentage of the population also eliminates the risk for selection bias. A limitation of the present study is that the cohort studied included only men, so our results may not apply to women. Furthermore, although the men in our study were followed for a maximum of 48 years, the average age at the end of follow-up is relatively young (56 years) and the etiologies of these PD diagnoses may differ from those occurring later in life.

## Conclusion

The present study reveals a significant association between increased ESR in adolescence and a lower risk of being diagnosed with PD later in life. Additional studies of patients with PD, looking at their immune responses, inflammatory activity during adolescence, and other diseases suffered and immunomodulatory treatments received during their lifetime are required.
